# Diversity and Seasonal Impact of *Acanthamoeba* Species in a Subtropical Rivershed

**DOI:** 10.1155/2013/405794

**Published:** 2013-12-30

**Authors:** Po-Min Kao, Ming-Yuan Chou, Chi-Wei Tao, Wen-Chien Huang, Bing-Mu Hsu, Shu-Min Shen, Cheng-Wei Fan, Yi-Chou Chiu

**Affiliations:** ^1^Department of Earth and Environmental Sciences, National Chung Cheng University, Chiayi 621, Taiwan; ^2^Department of Internal Medicine, Cheng Hsin Hospital, Taipei 112, Taiwan; ^3^Section of Respiratory Therapy, Cheng Hsin Hospital, Taipei 112, Taiwan; ^4^Division of Thoracic Surgery, Department of Surgery, Mackay Memorial Hospital, Mackay Medicine, Nursing, and Management College, Taipei 251, Taiwan; ^5^Department of Surgery, School of Medicine, National Yang Ming University, Taipei 112, Taiwan; ^6^General Surgery, Surgical Department, Cheng Hsin General Hospital, Taipei 112, Taiwan

## Abstract

This study evaluated the presence of *Acanthamoeba* species in the Puzih River watershed, which features typical subtropical monsoon climate and is located just above the Tropic of Cancer in Taiwan. The relationship between the seasonal and geographical distributions of *Acanthamoeba* species in this rivershed was also investigated. *Acanthamoeba* species were detected in water samples using the amoebal enrichment culture method and confirmed by PCR. A total of 136 water samples were included in this study, 16 (11.7%) of which contained *Acanthamoeba* species. Samples with the highest percentage of *Acanthamoeba* (32.4%) were obtained during the summer season, mainly from upstream areas. The identified species in the four seasons included *Acanthamoeba palestinensis* (T2), *Acanthamoeba* sp. IS2/T4 (T4), *Acanthamoeba lenticulata* (T5), *Acanthamoeba hatchetti* (T11), *Acanthamoeba healyi* (T12), and *Acanthamoeba jacobsi* (T15). The most frequently identified *Acanthamoeba* genotype was T4 (68.7%). *Acanthamoeba* genotype T4 is responsible for *Acanthamoeba keratitis* and should be considered for associated human health risk potential in the rivershed.

## 1. Introduction

*Acanthamoeba* has been isolated from a variety of environmental sources [1]. It is classified into three distinct morphological groups (I–III) based largely on the cyst morphology of the species and encompassing more than 25 nominal species [2, 3]. The developments in the subgenus classification and taxonomy of *Acanthamoeba* have been currently classified into 17 different genotypes (T1–T17) based on 18S rRNA gene sequencing by molecular techniques.* Acanthamoeba* causes serious human diseases, such as *Acanthamoeba *keratitis and granulomatous amoebic encephalitis [4]. *Acanthamoeba* genotype T4 is the most prevalent type causing disease in human [5], the main *Acanthamoeba* keratitis-related genotype worldwide.

*Acanthamoeba* has been distributed in different aquatic environments, climatic regions, and water quality conditions all over the world. The detection of *Acanthamoeba* in the temperate region, subtropical region, and tropical region was in the range of 7–94% [6–13], 3.6–21.2% [14–19], and 13.2–43.2% [20–26], respectively. In these regions, the water temperature differs according to the rivershed. Many human diseases are caused by pathogenic microorganisms, which are in turn influenced by a range of environmental factors. Seasonal changes may be correlated with the presence of *Acanthamoeba*. Although the influence of seasonal changes on free-living communities of *Amoebae* has been discussed in a number of reports [27–29], studies specific to *Acanthamoeba* in an aquatic environment are sparse, particularly in riversheds within a subtropical monsoon climate zone.

The main objectives of this study were to evaluate the effects of seasonal variations on the diversity of *Acanthamoeba* species and the presence/absence of these microbes in a subtropical river throughout a cycle of one year. Water quality parameters were also measured, the results of which are discussed with respect to the risk of infection in a given season.

## 2. Materials and Methods

### 2.1. Water Samples Collection and Water Quality Measurements

**Figure 1:**
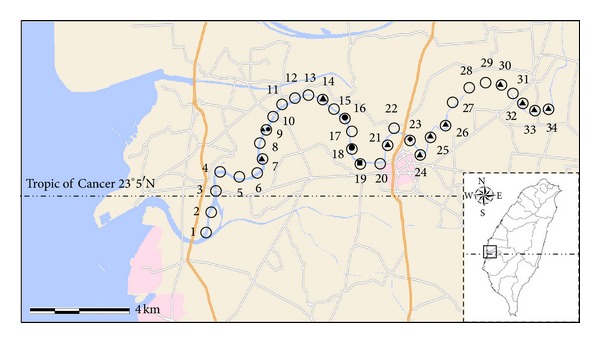
Sampling locations for *Acanthamoeba* positive in different seasons on the Puzih River in Taiwan. ○: sampling site; ■: spring; ▲: summer; ◆: autumn; ●: winter.

A total of 136 water samples were collected from 34 sampling locations along Puzih River (23°28′N, 120°13′E) in Southern Taiwan. The Puzih River lies within a subtropical monsoon region located near the Tropic of Cancer. A map of the study area with sampling locations is presented in Figure 1. A boat was used to take water samples at fixed distances between the ocean outflow and the headwaters. The river was divided into 3 groups: 11 upstream sites (24–34), 11 midstream sites (13–23), and 12 downstream sites (1–12). Samples were taken at each site once per quarter (three months). Sample collection was carried out between July 2009 and March 2010, covering all four seasons. For each sampling location, 2 L of water sample was placed into two sterile 1 L polypropylene bottles and stored at 4°C and transported to the laboratory for subsequent analyses within 24 h.

Physical water quality parameters were measured for each water sample on location at the time of sample collection. Water temperature and pH level were measured *in situ* using a thermometer and a portable pH meter (D-24E, Horiba Co., Kyoto, Japan). Turbidity was measured *in situ* using a ratio turbidimeter (Turb555, WTW, Germany). Additional water samples were taken from each sampling location in 300 mL sampling bags (Nasco Whirl-Pak, Salida, CA, USA) for microbiological water quality parameters. The samples were kept in coolers during transportation to the laboratory for subsequent analyses within 24 h. Heterotrophic bacteria were cultured on the M-(HPC) heterotrophic plate count agar base measured by the spread plate method (Methods 9215 C) [30]. In addition, total coliforms were measured by membrane filtration and a differential medium described in the standard method for the examination of water and wastewater (Methods 9222 B) [30]. The total coliform cultures were placed in m-Endo LES agar (Difco, USA) at 36 ± 1°C for 24 h before counting. The potential effect of water quality parameters on the presence/absence of *Acanthamoeba* species was evaluated using STATISTICA version 6.0 (StatSoft, Inc., Tulsa, OK, USA).

### 2.2. Sample Pretreatment

For each collected water sample, 2 L was concentrated via membrane filtration method using GN-6 Metricel mixed cellulose ester (MCE) membranes (0.45 *µ*m pore diameter, Pall, Port Washington, NY, USA). After filtration, the membranes were scraped, and the collected material was washed with 100 mL eluted fluid consisting of phosphate-buffered saline (PBS; 7.5 mM Na_2_HPO_4_, 3.3 mM NaH_2_PO_4_, and 108 mM NaCl, pH 7.2), 1% Tween 80, and 1% sodium dodecyl sulfate. The resulting solution was then transferred into two 50 mL conical centrifuge tubes and centrifuged at 2,600 ×g for 30 min. Subsequently, the top 45 mL supernatant fluid was removed, and the remaining 5 mL pellet was preserved with PBS at 4°C for further culture procedure and PCR analyses.

### 2.3. Culture and DNA Extraction for *Acanthamoeba* Species

The resulting pellet was transferred to a culture plate containing 1.5% nonnutrient agar (NNA) and a lawn of heat killed *Escherichia coli*; then, the plates were sealed and incubated at 30°C, relative humidity of 85% for 2 weeks. During incubation time, the *Acanthamoeba* candidates were transferred onto new NNA plates containing a lawn of heat killed *E. coli* 1–3 times to avoid fungal contamination. The transfer times were determined by considering the fungal growth situation. The *Acanthamoeba* candidates on the plate were transferred to 5 mL glass tubes with PYG medium consisting of 2% proteose peptone, 0.2% yeast extract, 0.1 M glucose, and 1% Gibco antibiotic-antimycotic (Cat. No. 15240-06, USA) and incubated at 32°C for 3-4 days. The samples that yielded *Acanthamoeba* species were harvested and then were confirmed by molecular taxonomic identification method.

DNA extraction was done with the concentrated pellet using MagNA Pure LC instruments (Roche, USA) with MagNA Pure LC DNA isolation kits III (Roche, USA) following automated mode specified by the kit manufacturer's instructions manual. The resulting solution was analyzed for the presence of *Acanthamoeba* species specific genes with PCR assays.

### 2.4. PCR Conditions, Gel Electrophoresis, and Gene Sequence Analysis

The PCR assay genus-specific primers' sets JDP1 and JDP2 used in this study were designed for *Acanthamoeba* genotyping as previously described [31]. They were established as the following sequences: 5′-GGCCCAGATCGTTTACCGTGAA-3′ and 5′-TCTCACAAGCTGCTAGGGGAGTCA-3′, to amplify a 450 bp fragment of the 18S rDNA stretch *Acanthamoeba*-specific amplimer ASA.S1 of *Acanthamoeba* genotypes.

PCR reaction solution was prepared with 5 *μ*L of the DNA templates together with the PCR mixture to create a total volume of 25 *μ*L. The PCR mixture had 0.5 *μ*L PCR buffer (25 Mm MgCl_2_), 0.5 *μ*L dNTP Mix (10 Mm of each dNTP), 1 *μ*L each of the oligonucleotide primers, and 0.1 *μ*L VioTaqTM DNA Polymerase (VIOGENE, 5 U/*μ*L), as well as DNase-free deionized water. Cycling conditions were as follows: 95°C for 5 min for the initial denaturation step, followed by 35 cycles of 15 s at 95°C for denaturation, 15 s at 62°C for annealing, 3 s at 72°C for extension, and a final extension at 72°C for 10 min.

Negative DNA controls (template DNA replaced with distilled water) and positive controls (*Acanthamoeba lenticulata* ATCC30841) and sample DNA were analyzed in triplicates during each PCR run. PCR products of *Acanthamoeba* species were detected with gel electrophoresis on a 2% agarose gel (Biobasic Inc., Markham, ON, Canada) performed with 5 *μ*L of the reaction solution. The DNA fragments were confirmed using ethidium bromide staining (0.5 *μ*g/mL, 10 min). A 100 bp DNA ladder was used as a DNA size marker. The sequence analysis was done using a Bio-Dye terminator cycle sequencing kit (Applied Biosystems, Carlsbad, CA, USA). Phylogenetic construction produced gene trees by using neighbor-joining distance trees with a generation of 1,000 bootstrapped replicates. The 18S rRNA gene sequences were assigned to the GenBank database in order to allow BLAST searching and alignment using the MEGA software program version 5.0 (Mega Software, Tempe, AZ, USA). The highest percentage similarity was taken to identify the species.

## 3. Results and Discussion

### 3.1. Occurrence and Seasonal Distribution of *Acanthamoeba* Species in a Subtropical River

A total of 136 water samples were collected in this study. The detection results of *Acanthamoeba* species from river waters are shown in Table 1. *Acanthamoeba* species were detected in 16 of the 136 samples (11.7%). The detection results for *Acanthamoeba* species were further analyzed with respect to detection rates by four sampling seasons, and the results are presented in Table 1. Seasonal detection rates of *Acanthamoeba* species were the spring (2.9%), summer (32.4%), autumn (2.9%), and winter (8.8%). The greatest percentage of *Acanthamoeba* species was detected during the summer. The results support a previous report that *Acanthamoeba* species were most prevalent in late summer in the aquatic environment [32, 33]. The geographic distribution and occurrence of *Acanthamoeba* species along Puzih River were further compared by sampling seasons, and the results are presented in Figure 1. Geographic distribution was determined by the detection of *Acanthamoeba* species throughout the year. During the summer, *Acanthamoeba* was found in the upstream (7/11, 63.6%), midstream (2/11, 18.1%), and downstream (2/12, 16.6%) areas. In contrast, only single detection was obtained in the midstream areas during spring and autumn, respectively. Detection rates in the winter were 18.1% (2/11) and 8% (1/12) in the midstream and downstream areas, respectively. In Taiwan, rainfall mainly occurs in summer, with occasional typhoon and frequent thunderstorm. In the Puzih River, the total amount of rainfall in summer makes up roughly 90% of the annual rainfall amount for the study region. As shown in Figure 1, sampling locations with *Acanthamoeba* species detection principally occurred in the upstream areas. It is possible that water contaminants (such as livestock wastewater and sewage) may be carried into the river after rainfall [34, 35] or riverbed sediment underwent seasonal rainfall in summer [32, 33] that these sediments supported microbial growth [36]. In addition, weather events were found to play a major role in the presence/absence of *Acanthamoeba* species in the rivershed, with such changes probably due to resuspension of *Acanthamoeba* species from riverside or riverbed sediment by rainfall or wind action and input from the rivershed via runoff [32].

### 3.2. Identification of *Acanthamoeba* Species in the Four Seasons

The sixteen *Acanthamoeba*-positive samples detected by PCR were subjected to DNA sequencing for species identification. This was done by neighbor-joining analysis, in which sample strains were compared with reference *Acanthamoeba* strains from the NCBI GenBank to determine likelihood of sample content being specific strains. The results are shown in Table 1. The identified *Acanthamoeba* species (genotypes) were *Acanthamoeba palestinensis* (T2), *Acanthamoeba *sp. IS2/T4 (T4), *Acanthamoeba lenticulata* (T5), *Acanthamoeba hatchetti* (T11), *Acanthamoeba healyi* (T12), and *Acanthamoeba jacobsi* (T15). The most frequently identified *Acanthamoeba* genotype was T4 (*n* = 11), followed by T2, T5, T11, T12, and T15 which were each detected once. These genotypes were associated with *Acanthamoeba* keratitis, granulomatous amoebic encephalitis, and chronic granulomatous lesions of the skin [2, 3, 37, 38].

For the seasonal distribution results, sample A19 was detected *Acanthamoeba *sp. IS2/T4 (NCBI EU934065.1) in spring, which is a widely distributed genotype in water samples from Iran, where it was isolated from *Acanthamoeba* keratitis patients [37]. In summer, samples B7, B9, B14, B24, B25, B26, B30, B32, and B33 were identified as* Acanthamoeba *sp. IS2/T4 (NCBI EU934065.1). Sample B21 was identified as *Acanthamoeba hatchetti* (NCBI AF251939.1), which causes *Acanthamoeba* keratitis [2, 3] and sample B34 was identified as* Acanthamoeba palestinensis* (NCBI GU597012.1), which has ever been isolated from hot springs in Taiwan [16]. In autumn, sample C23 was identified as similar to *Acanthamoeba lenticulata* (ATCC 30841) isolated from swimming pool in France [39, 40]. In winter, sample D9 was identified as* Acanthamoeba *sp. IS2/T4, sample D16 was identified as *Acanthamoeba jacobsi *(NCBI GU573872.1) whose survival range includes cold streams, springs, and hot springs in Taiwan [16], and sample D18 was identified *Acanthamoeba healyi* (NCBI AF019070.1), which was first isolated from granulomatous amoebic encephalitis patient in Barbados [40].

Quarterly monitoring revealed that the diversity of *Acanthamoeba* species varied according to season and sampling location and evidence further indicated that seasonal effects influence the diversity of *Acanthamoeba* species in the rivershed. In addition, the results of this study mainly indicate that *Acanthamoeba* species genotype T4 accounted for 68.7% of all positive samples. The highest proportion of *Acanthamoeba* keratitis cases was associated with the T4 genotype, possibly due to its greater virulence and greater transmissibility with respect to other genotypes. Therefore, the presence of pathogenic *Acanthamoeba* should be considered a potential health threat associated with human activities in Puzih River watershed.

### 3.3. Relations between *Acanthamoeba* Presence and Water Quality Parameters

Water quality parameters were compared with respect to *Acanthamoeba* species detection results. The range, mean, and standard deviation of water quality parameters in four seasons are included in Table 2. Statistical analyses were performed to detect differences (Mann-Whitney *U* test) and correlation coefficients (Spearman rank test) in water quality parameters between *Acanthamoeba*-positive and negative samples. Results of the above nonparametric tests are presented in Table 3. No significant difference and correlation were observed between the presence/absence of *Acanthamoeba* species for all the water quality parameters in the total water samples. For sampling season impact, no significant difference and correlation were observed between the presence/absence of *Acanthamoeba* species for all the water quality parameters in spring, autumn, and winter. However, significant differences were observed only between the presence/absence of *Acanthamoeba* species and turbidity (*P* < 0.05), and significant correlation were found only between the presence/absence of *Acanthamoeba* species, heterotrophic plate count (HPC), and turbidity (*P* < 0.05) in summer. The correlations and differences between turbidity and *Acanthamoeba* species demonstrated that sediments supported microbial growth [36]. The HPC from the natural inhabitants in the aquatic environment may cause the predation of* Acanthamoeba* species [16]. It explains why the presence/absence of *Acanthamoeba* species had a significant correlation with HPC. The results suggested that there were seasonal characteristics over water quality parameters in the studied river, and the seasonal variations may play an important role in the presence/absence of *Acanthamoeba* species.

## 4. Conclusions

The total detection rate was 11.7% for *Acanthamoeba *species in a subtropical river. For seasonal monitoring results, *Acanthamoeba* species were detected mainly in the upstream areas during summer. The most mainly identified *Acanthamoeba *species genotypes were T4, which was associated with *Acanthamoeba* keratitis. The significant difference (turbidity) and correlation (HPC and turbidity) were found between water quality parameters and the presence/absence of *Acanthamoeba* species in summer.

## Figures and Tables

**Table 1:** Distribution of *Acanthamoeba* species by different seasons of isolation.

Sampling season	Sample size	Number of detection for *Acanthamoeba *	Percent of detection for *Acanthamoeba *	Positive sample no.	Species name	Genotypes
Spring	34	1	2.9%	A19	*Acanthamoeba* sp. IS2/T4 (NCBI EU934065.1)	T4
Summer	34	11	32.4%	B7	*Acanthamoeba* sp. IS2/T4 (NCBI EU934065.1)	T4
				B9	*Acanthamoeba* sp. IS2/T4 (NCBI EU934065.1)	T4
				B14	*Acanthamoeba* sp. IS2/T4 (NCBI EU934065.1)	T4
				B21	*Acanthamoeba hatchetti* (NCBI AF251939.1)	T11
				B24	*Acanthamoeba* sp. IS2/T4 (NCBI EU934065.1)	T4
				B25	*Acanthamoeba* sp. IS2/T4 (NCBI EU934065.1)	T4
				B26	*Acanthamoeba* sp. IS2/T4 (NCBI EU934065.1)	T4
				B30	*Acanthamoeba* sp. IS2/T4 (NCBI EU934065.1)	T4
				B32	*Acanthamoeba* sp. IS2/T4 (NCBI EU934065.1)	T4
				B33	*Acanthamoeba* sp. IS2/T4 (NCBI EU934065.1)	T4
				B34	*Acanthamoeba palestinensis* (NCBI GU597012.1)	T2
Autumn	34	1	2.9%	C23	*Acanthamoeba lenticulata* (ATCC 30841)	T5
Winter	34	3	8.8%	D9	*Acanthamoeba *sp. IS2/T4 (NCBI EU934065.1)	T4
				D16	*Acanthamoeba jacobsi* (NCBI GU573872.1)	T15
				D18	*Acanthamoeba healyi *(NCBI AF019070.1)	T12

Total	136	16	11.7%			

**Table 2:** Ranges, mean, and standard deviation of water quality parameters during different seasons.

Water quality parameter	Heterotrophic plate counts (CFU/mL)		Total coliforms (CFU/100 mL)		Turbidity (NTU)		Temperature (°C)		pH value	
	Range	Mean ± SD	Range	Mean ± SD	Range	Mean ± SD	Range	Mean ± SD	Range	Mean ± SD
Spring	2.3 × 10^2^–1.0 × 10^5^	2.2 × 10^4^ ± 2.41 × 10^4^	0–9.8 × 10^4^	2.5 × 10^4^ ± 2.71 × 10^4^	25–151	95 ± 33.4	19.8–22.9	21.3 ± 0.74	7.64–8.23	7.9 ± 0.17
Summer	2.1 × 10^3^–1.1 × 10^6^	6.9 × 10^4^ ± 1.56 × 10^4^	12–9.9 × 10^2^	1.7 × 10^2^ ± 1.69 × 10^2^	34–206	86 ± 46.7	31.0–33.2	31.9 ± 0.49	7.67–8.09	7.9 ± 0.11
Autumn	2.9 × 10^2^–3.1 × 10^5^	2.5 × 10^4^ ± 5.23 × 10^4^	3.3 × 10^2^–7.4 × 10^4^	2.5 × 10^4^ ± 2.51 × 10^4^	25–471	103 ± 73.9	25–25.3	25.1 ± 0.09	7.71–8.40	7.9 ± 0.14
Winter	18.5–6.2 × 10^5^	5.0 × 10^4^ ± 1.34 × 10^5^	22–1.4 × 10^4^	5.2 × 10^3^ ± 4.30 × 10^3^	40–155	83 ± 25.7	18.4–22.9	20.0 ± 1.28	8.11–8.76	8.4 ± 0.18

Total	18.5–1.1 × 10^6^	4.4 × 10^4^ ± 1.14 × 10^5^	0–9.8 × 10^4^	1.2 × 10^4^ ± 2.06 × 10^4^	25–471	91 ± 48.0	18.4–33.2	25.2 ± 5.01	7.64–8.76	8.0 ± 0.27

**Table 3:** Nonparametric test results for difference and correlation for *Acanthamoeba* in terms of water quality parameters.

Water quality parameter	Spring		Summer		Autumn		Winter		Total	
	Mann-Whitney *U* test	Spearman rank test	Mann-Whitney *U* test	Spearman rank test	Mann-Whitney *U* test	Spearman rank test	Mann-Whitney *U* test	Spearman rank test	Mann-Whitney *U* test	Spearman rank test
Heterotrophic plate counts (CFU/mL)	*P* = 1.000	*R* = 0.292 *P* = 0.092	*P* = 0.112	*R* = −0.509 *P* = 0.002*	*P* = 1.000	*R* = −0.044 *P* = 0.803	*P* = 0.092	*R* = 0.293 *P* = 0.092	*P* = 0.174	*R* = 0.194 *P* = 0.176
Total coliforms (CFU/100 mL)	*P* = 1.000	*R* = 0.097 *P* = 0.582	*P* = 0.623	*R* = 0.112 *P* = 0.527	*P* = 1.000	*R* = −0.079 *P* = 0.653	*P* = 0.379	*R* = 0.152 *P* = 0.387	*P* = 0.942	*R* = −0.010 *P* = 0.943
Turbidity (NTU)	*P* = 1.000	*R* = −0.204 *P* = 0.247	*P* = 0.049*	*R* = −0.349 *P* = 0.042*	*P* = 1.000	*R* = 0.275 *P* = 0.115	*P* = 0.164	*R* = −0.242 *P* = 0.167	*P* = 0.163	*R* = −0.199 *P* = 0.165
Temperature (°C)	*P* = 1.000	*R* = 0.2932 *P* = 0.092	*P* = 0.663	*R* = −0.041 *P* = 0.814	*P* = 1.000	*R* = −0.231 *P* = 0.188	*P* = 0.143	*R* = 0.255 *P* = 0.145	*P* = 0.204	*R* = 0.181 *P* = 0.207
pH value	*P* = 1.000	*R* = 0.097 *P* = 0.582	*P* = 0.438	*R* = 0.038 *P* = 0.828	*P* = 1.000	*R* = 0.062 *P* = 0.725	*P* = 0.241	*R* = −204 *P* = 0.247	*P* = 0.174	*R* = −0.194 *P* = 0.176

Note: *P < 0.05.
